# Cancer, more than a “COVID-19 co-morbidity”

**DOI:** 10.3389/fonc.2023.1107384

**Published:** 2023-03-13

**Authors:** Chinmay T. Jani, Robert T. Schooley, Rana R. Mckay, Scott M. Lippman

**Affiliations:** ^1^ Department of Medicine, Mount Auburn Hospital, Cambridge, MA, United States; ^2^ Department of Medicine, Harvard Medical School, Boston, MA, United States; ^3^ Division of Hematology-Oncology, Department of Medicine, University of California San Diego, La Jolla, CA, United States

**Keywords:** Cancer, COVID-19, risk factor, pharmacological management, comorbidity

## Abstract

Patients with cancer represent a particularly vulnerable population at risk of adverse outcomes related to COVID-19. Collectively, the initial studies, including patients with and without cancer, confirmed that patients with cancer had a higher risk of complications and death related to COVID-19. Subsequent studies on patients with COVID-19 and cancer investigated patient and disease-related factors associated with COVID-19 severity and morality. Multiple interconnected factors include demographics, comorbidities, cancer-associated variables, treatment side effects, and other parameters. However, there is a lack of clarity on the contributions of any one factor. In this commentary, we deconvolute the data of specific risk factors associated with worse outcomes due to COVID-19 in cancer patients and focus on understanding the recommended guidelines to mitigate COVID-19 risk in this vulnerable population. In the first section, we highlight the key parameters, including age and race, cancer status, type of malignancy, cancer therapy, smoking status and comorbidities that impact outcomes for cancer patients with COVID-19. Next, we discuss efforts made at the patient, health system, and population levels to mitigate the effects of the ongoing outbreak for patients with cancer, including (1) screening, barrier and isolation strategies (2), Masking/PPE (3), vaccination, and (4) systemic therapies (e.g., evusheld) to prevent disease onset in patients. In the last section, we discuss optimal treatment strategies for COVID-19, including additional therapies for patients with COVID-19 and cancer. Overall, this commentary focuses on articles with high yield and impact on understanding the evolving evidence of risk factors and management guidelines in detail. We also emphasize the ongoing collaboration between clinicians, researchers, health system administrators and policymakers and how its role will be important in optimizing care delivery strategies for patients with cancer. Creative patient-centered solutions will be critical in the coming years, post the pandemic.

## Highlights

Patients with cancer represent a particularly vulnerable population at risk of adverse outcomes related to COVID-19. Since the beginning of the pandemic, multiple studies, including extensive cohort studies, have tried to investigate this increased burden for patients with cancer. However, as the dust settles down, there is a need for granular data on risk assessment and management to better guide the therapy of these patients. In this commentary, we deconvolute the data of specific risk factors associated with worse outcomes due to COVID-19 in cancer patients and focus on understanding the recommended guidelines to mitigate COVID-19 risk in this vulnerable population. We highlight the key parameters, including age and race, cancer status, type of malignancy, cancer therapy, smoking status and comorbidities that impact outcomes for cancer patients with COVID-19. We also review the latest guidelines for the inpatient and outpatient management of COVID-19 for patients with cancer. For better visual presentation and understanding, we have also summarized our findings in graphical format throughout the manuscript. Overall, this commentary focuses on articles with high yield and impact on understanding the evolving evidence of risk factors and management guidelines in detail.

## 1 Introduction

Patients with cancer represent a particularly vulnerable population at risk of adverse outcomes related to COVID-19. There are multiple interconnected factors, including demographics, comorbidities, cancer-associated variables, treatment side effects, and other parameters that impact COVID-19 risk and severity in cancer. A series of studies have investigated the significance of various clinical risk factors on COVID-19 outcomes. However, there is a lack of clarity on the contributions of any one factor to COVID-19 risk (1). Risk assessment is critical given that COVID-19 prevention and treatment recommendations have varied depending on individual patients’ risk. With the ongoing pandemic, care delivery for cancer patients, including screening, medical procedures, treatment, and clinical trials, has been broadly impacted. Strategies to continue care delivery safely and efficiently during the pandemic have required multilevel infrastructure modifications. Changes have been implemented at the level of individual patient and care team members, clinical operations, institutional operations, and more extensive state/federal policy and guidelines (2). In this commentary, we deconvolute the data of specific risk factors associated with worse outcomes due to COVID-19 in cancer patients and focus on understanding the recommended guidelines to mitigate COVID-19 risk in this vulnerable population.

## 2 Data source

Given the nature of the pandemic with a need for immediate access to the data, much of the information which exists to help inform prognosis and risk factors in cancer patients is derived from retrospective studies. Cancer registries of patients infected with SARS-Cov-2 emerged during the first wave of the pandemic to address evolving questions about the outcomes of cancer patients with COVID-19. The COVID-19 and Cancer Consortium (CCC19) is a retrospective registry to investigate COVID-19 outcomes in patients with cancer and has spearheaded several landmark works looking at risk factors in patients with Cancer and COVID-19. Additional data are derived from UKCCMP (UK), OnCOVID (UK, Italy, Spain), DOCC (Dutch Oncology COVID-19 Consortium), GCO-002 CACOVID (French), ASCO registry (USA), N3C (NCATS’ National COVD Cohort Collaborative), and Teravolt registries and data sets (3, 4). Retrospective data have biases, issues of missing data, lack of control, or comparative arm. However, this data has been very informative in a time of need to understand variables influencing COVID-19 outcomes. This commentary focuses on articles with high yield and impact to understand the risk factors and management guidelines in detail.

## 3 Key risk factors for severe COVID-19 infection in cancer patients

### 3.1 Cancer as a risk factor

**Figure 1:**
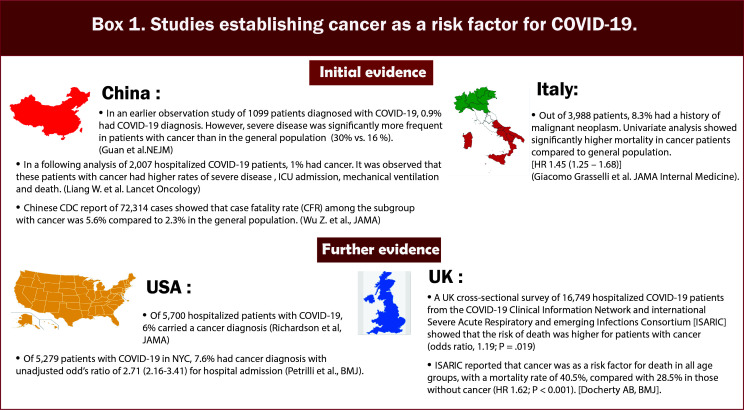
Box-1 Studies establishing cancer as a risk factor for COVID-19.

**Figure 2:**
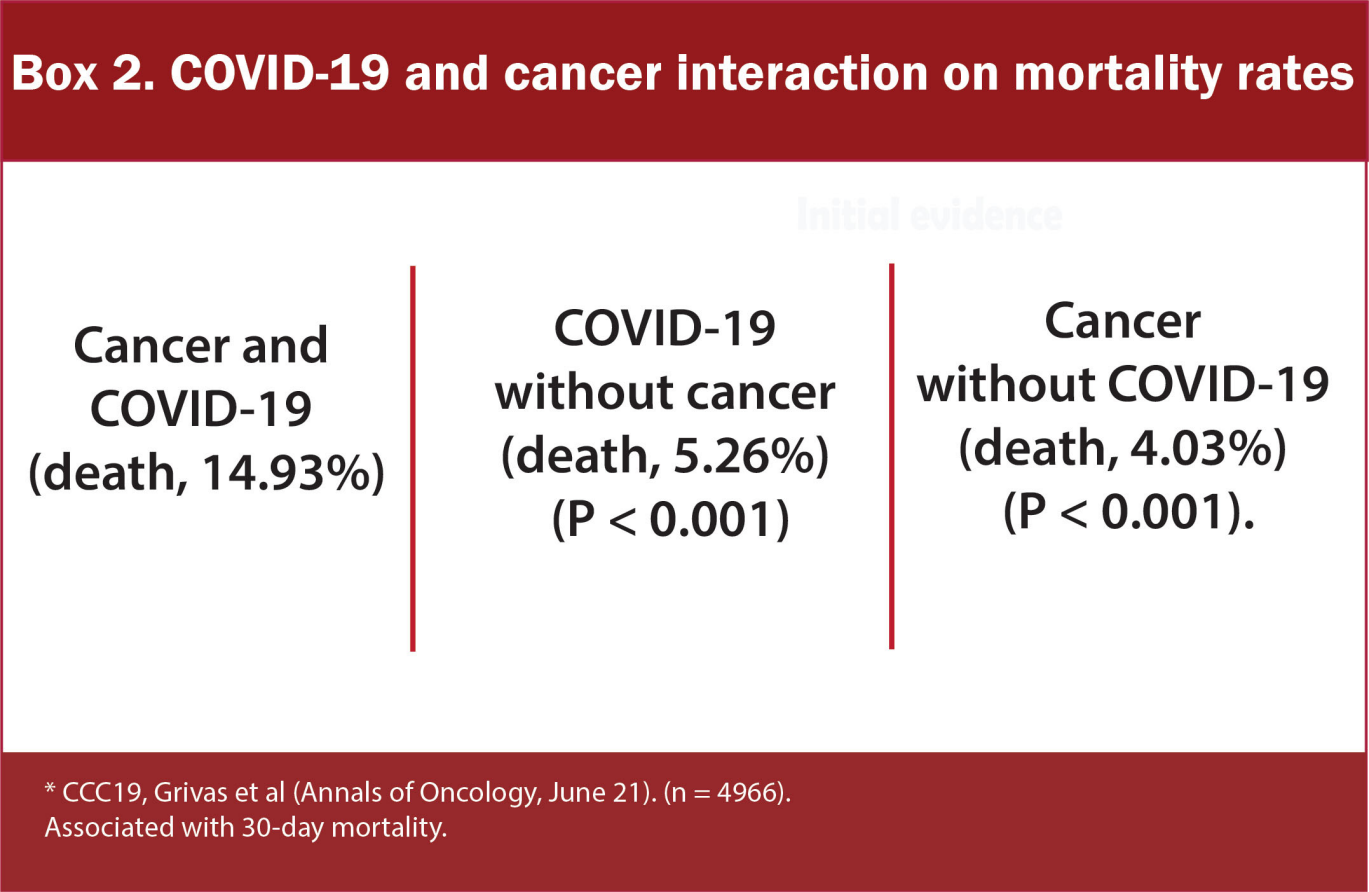
Box-2 COVID-19 and cancer interaction on mortality rates.

**Figure 3:**
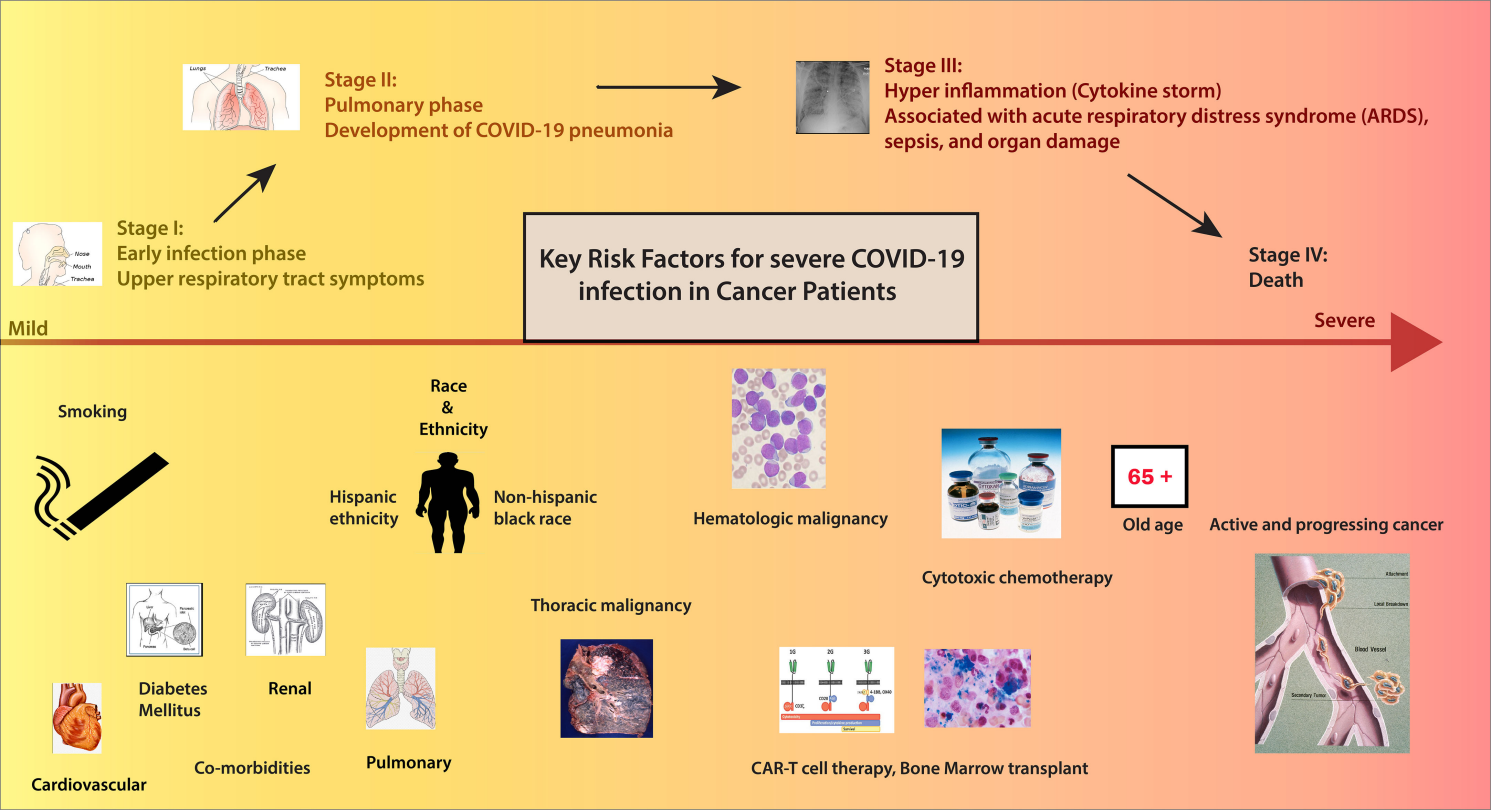
Risk factors of patients with Cancer in COVID-19. Credits for Active cancer image: Jane Hurd. National Cancer Institute.

In the initial reports of COVID-19 from China, patients with cancer had a higher infection rate than the general population (5–7) (**Figure 1**/Box 1). In these studies, patients with cancer made up a small proportion (0.9%) of COVID-19 cases. In an early observational study of 1099 patients diagnosed with COVID-19 in Wuhan, Intensive Care Unit (ICU) admission was required in 5.0%, use of mechanical ventilation in 2.3%, and death occurred in 1.4%. Even though only 0.9% had cancer diagnoses, the severe disease was significantly more frequent in patients with cancer than in the general population (30% vs. 16%) (6). In the following analysis of 2,007 hospitalized COVID-19 patients from China, 18 (1%) had a history of cancer. Cancer patients had more severe diseases with higher rates of ICU admission, mechanical ventilation, and death (7). According to a report from the Chinese CDC of 72,314 cases, the case fatality rate (CFR) among the subgroup with cancer was 5.6% compared with 2.3% in the general population (8). Multiple studies have corroborated these findings (6, 7). As the pandemic’s epicenter shifted, reports from the US and the UK demonstrated that cancer is an independent risk factor for COVID-19 disease severity (9–11) (**Figure 2**/Box 2). A UK cross-sectional survey of 16,749 hospitalized COVID-19 patients from the COVID-19 Clinical Information Network and International Severe Acute Respiratory and Emerging Infections Consortium [ISARIC] showed that the risk of death was higher for patients with cardiac, pulmonary, and kidney disease, as well as cancer after correction for demographic variables. Patients with a history of cancer had an increased risk of death due to COVID-19 compared with those without a cancer history (odds ratio, 1.19; P = 0.019). ISARIC reported that cancer was a risk factor for death in all age groups, with a mortality rate of 40.5%, compared with 28.5% in those without cancer (HR 1.62; P < 0.001) (12). Collectively, these initial studies, including patients with and without cancer, confirmed that patients with cancer had a higher risk of complications and death related to COVID-19. Subsequent studies conducted on patients with COVID-19 and cancer investigated patient and disease characteristics associated with COVID-19 severity and morality. We highlight the key parameters, including age and race, cancer status, type of malignancy, cancer therapy, smoking status and comorbidities that impact outcomes for cancer patients with COVID-19. **Figure 3** and **Table 1** illustrates the major risk factors of severe COVID-19 in cancer patients.

**Table 1:** Comparison of key risk factors in different observational studies.

	Grivas et al. *CCC-19* (n=4966) * OR (95% CI)	Cortellini et al. *OnCOVID* (n=2795) # HR (95% CI)	Sharafeldin et al. *N3C* (n=38,614)$ HR (95% CI)
Age	1.75 (1.59 – 1.93) **	1.58 (1.33 – 1.89) ##	1.99 (1.27 – 3.10) $$
Sex	Male [1.46 (1.20 – 1.77)] ref female	Female [0.92 (0.78 – 1.09)] ref male	Female [0.90 (0.83-0.98)], ref male
Race	(Non-Hispanic black) 1.38 (1.09 – 1.755)	N/A	Black or African American 0.82 (0.73 – 0.92)
Ethnicity - Hispanic	1.31 (0.96 -1.80)	N/A	0.98 (0.74 – 1.30)
Smoking	1.20 (0.98 – 1.46) **	N/A	1.12 (0.99 – 1.28)
ECOG performance status ≥2	4.48 (3.34-6.00)	N/A	
Comorbidities		>/= 2 [1.25 (1.06 – 1.46)] (ref 0 – 1)	
Charlson-Comorbidity index	—–		CCI >/= 4 (2.04 (1.79 – 2.33)
Pulmonary comorbidities	1.34 (1.09 – 1.66)		N/A
DM	1.23 (1.00 – 1.50)		N/A
Cardiovascular	1.17 (0.95 – 1.43)		N/A
Renal disorders	1.31 (1.05 – 1.63)		N/A
Type of cancer			
Hematological malignancy	1.44 (1.10 – 1.87)	0.98 (0.71 – 1.36) ref Breast cancer	1.15 (1.02 – 1.29)
Cancer status			
Active and progressing	2.88 (2.13 – 3.90)	1.64 (1.36 – 1.99)	N/A
Anticancer Treatment		Any anticancer therapy [1.33 (1.13 – 1.56)] vs. no anticancer therapy	
Cytotoxic therapy	1.61 (1.15-2.24)		1.52 (1.13 – 2.06)
Anti-COVID-19 management		Any COVID-19 therapy [1.07 (0.92 – 1.25)]	
Remdesivir, yes versus no	1.55 (1.10-2.18)		0.98 (0.84 – 1.14)
Corticosteroids alone, yes versus no	1.86 (1.35-2.56)		Dexamethasone 0.8 (0.7 – 0.9)

*Associated with 30-day mortality. ** Smoking status, ever versus never (13).

$Associated with 1-year all-cause mortality $$ (Age >65 vs. 18-29 as reference) (4).

#Associated with risk of death at 14 days ## (Age >/= 65 vs <65 as reference) (14). N/A, Not Available.

### 3.2 Age and race

**Figure 4:**
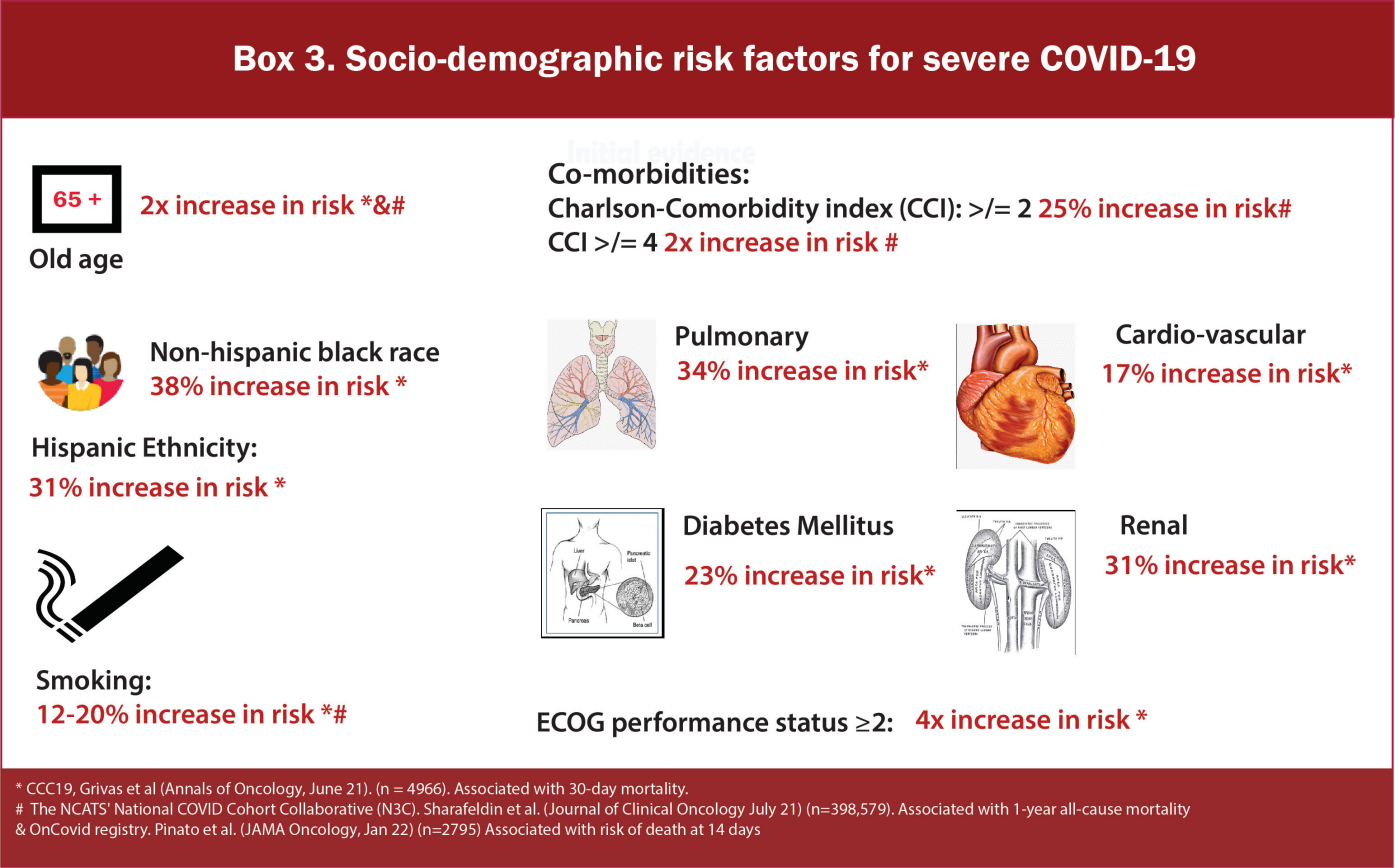
Box-3 Socio-demographic risk factors for severe COVID-19.

Several studies have demonstrated that age, non-Hispanic black race, and Hispanic ethnicity are negative predictors of Covid-19 outcomes in patients with cancer (15) (**Figure 4**/Box 3). Among patients with a recent cancer diagnosis, African American individuals had a significantly higher risk for COVID-19 infection than White individuals (16). Even after adjustment for co-morbidities and disease-related factors, these demographic variables remained independent predictors of worse outcomes in cancer patients, paralleling data derived from general cohorts of patients without cancer. As we better understand the impact of these demographics on COVID-19 outcomes, it is critical to factor in the impact of social determinants of health, transportation, access to care, and virtual resources on outcomes. We need to develop strategies to bridge disparities for minority and disparate populations and ensure equitable COVID-19 and cancer-directed care during the pandemic.

### 3.3 Cancer status

**Figure 5:**
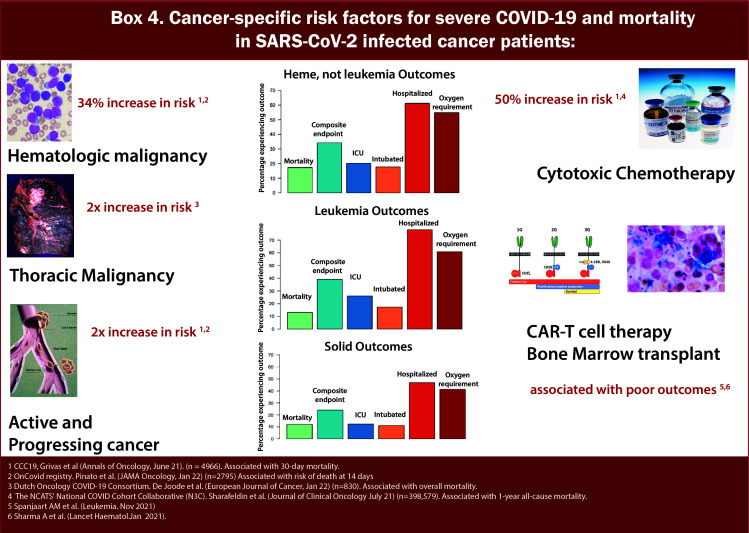
Box-4 Cancer-specific risk factors for severe COVID-19 mortality in SARS-CoV-2 infected cancer patients.

Various groups have further studied the impact of cancer status on COVID-19 outcomes (**Figure 5**/Box 4). While the definition of active cancer varied across a series of reports, generally, it has been defined as a metastatic disease in patients with solid tumors and/or recent cancer treatment (<90 days from diagnosis of COVID-19) (17, 18). In the CCC-19 report, patients with active or progressing cancer were found to have higher 30-day mortality [OR 2.88 (2.13-3.90)] as compared to patients in remission or having no evidence of disease (13). A study from the UKCCMP group on patients with active cancer with COVID-19 showed a mortality rate of 38% (966/2515) (19). Additional studies have also confirmed that patients with distant metastases or recurrent disease are at increased risk of worse COVID-19 outcomes than patients without such conditions (20). In aggregate, these data suggest that patients with active cancer are particularly vulnerable to adverse COVID-19 outcomes and warrant special considerations for risk reduction strategies.

### 3.4 Type of malignancy

**Figure 6:**
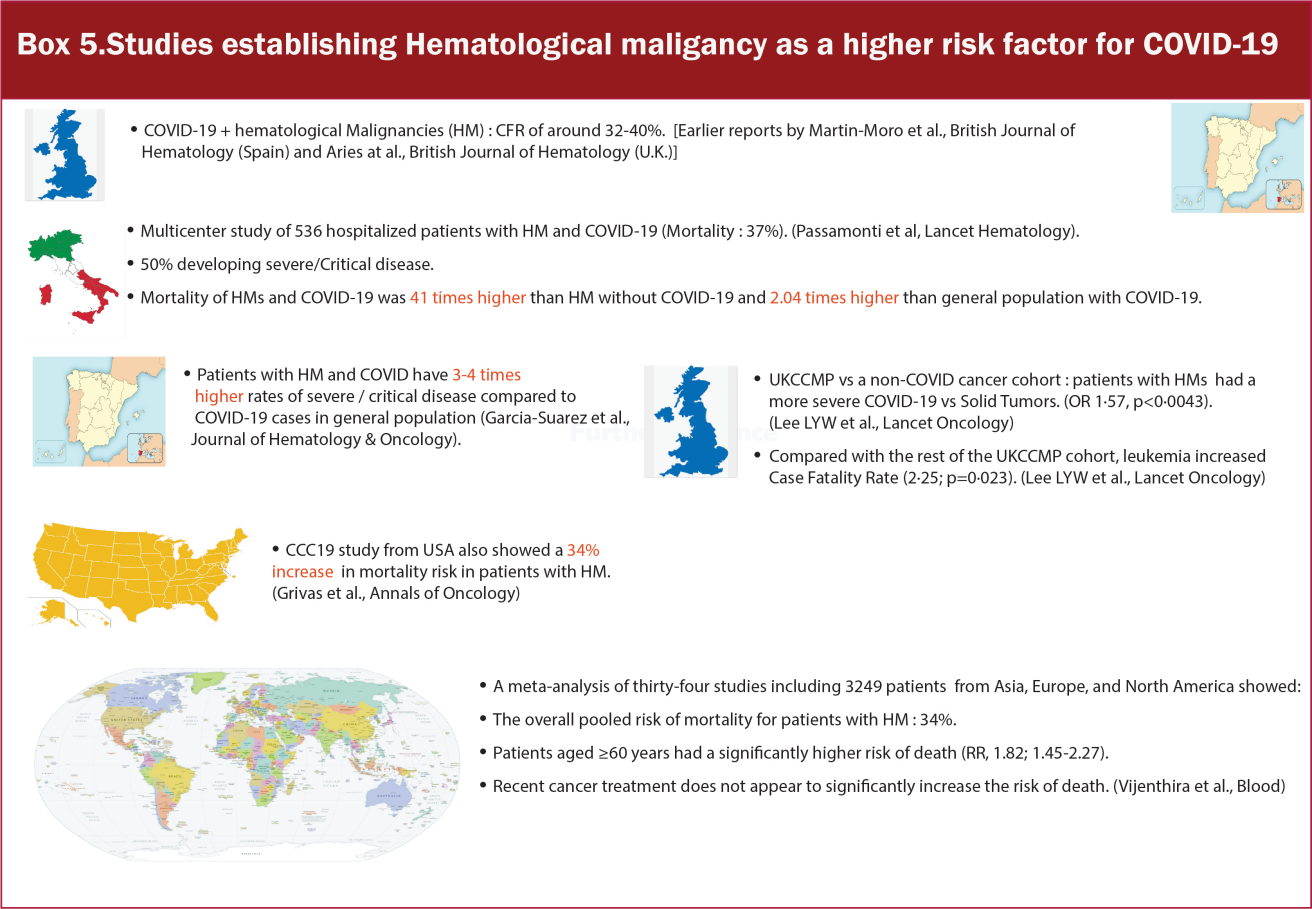
Box-5 Studies establishing hematological malignancy as a higher risk factor for COVID-19.

Many different registries and consortiums have shown increased severity and mortality in patients with hematologic malignancies (HM) (**Table 2** and **Figure 6**/Box 5). HM leads to a constellation of immune alterations that could compromise the immune response caused by SARS-CoV-2 (32). Studies have demonstrated that patients with HM have an increased viral load compared to non-cancer patients and patients with solid tumors (33). Additionally, patients with HMs are observed to have a higher rate of severe, critical COVID infection and significantly increased mortality risk than those with solid tumors (17, 21–25, 34). They are at increased risk of mortality regardless of the time of diagnosis or concurrent receipt of any specific therapy (chemotherapy, targeted therapy, or immunotherapy). Earlier reports from Spain and the UK on the impact of COVID-19 on HM showed CFR of around 32-40% (35, 36). In a multicenter study of 536 hospitalized patients in Italy with HM and COVID-19, mortality was approximately 37%, with 50% developing severe/Critical disease. On calculating the standardized mortality ratio based on observed and expected death of this population in Italy, it was found that mortality was 41 times higher in patients with HM and COVID-19 compared to individuals with HM without COVID-19 and 2 times higher than the general population with COVID-19. Amongst HM, a diagnosis of AML (HR 3.49, 1·56–7·81), indolent non-Hodgkin Lymphoma (2·19, 1·07–4·48), aggressive non-Hodgkin lymphoma (2·56, 1·34–4·89), or plasma cell neoplasms (2·48, 1·31–4·69) were associated with worse overall survival. It was also observed that there was no association between overall survival and time since HM diagnosis or last treatment for HMs, indicating that they are at high risk of mortality regardless of whether they have a recent disease or are on a specific therapy or both (27). At the same time, a population-based registry study from Spain showed that patients with HM and COVID-19 have 3-4 times higher rates of severe/critical disease compared to COVID-19 cases in the general population (26). A subsequent analysis of UKCCMP compared to a non-COVID-19 cancer cohort *via* the UK Office of National Statistics showed patients with HMs had more severe COVID-19 vs. Solid Tumors (OR 1·57, p<0·0043). Compared with the rest of the UKCCMP cohort, leukemia increased CFR (2·25; p=0·023). After correction for age and sex, patients with HMs who had recent chemotherapy within 4 weeks of COVID-19 had a 2-fold increased death risk (24). A meta-analysis of 34 studies including 3249 patients demonstrated that the overall pooled risk of mortality for patients with HM was approximately 34% (15).

**Table 2:** Outcomes in hematological malignancies.

Studies	Overall hematological malignancies	Leukemia	Lymphoma	Myeloproliferative neoplasms
Jee J et al. (21)	HR of severe or critical COVID-19 disease 1.90, 95% CI 1.30 – 2.80	**AML:** 87.5% of patients developed critical illness	Not reported	Not reported
Robilotti et al. (22)	HR of hospitalization 2.49, 95% CI 1.35-4.67. HR of severe respiratory illness 1.79, 95% CI 0.97 – 3.32.	Not reported	Not reported	Not reported
Williamson E J et al. (23)	HR for COVID-19-related death of 2.80 (95% CI 2.08 – 3.78) in the patient with a HM diagnosis 1 year before COVID-19 vs. none. 95% HR for COVID-19-related death of 1.61 (95% CI 1.39 – 1.87) if HM diagnosed >/=5 years ago vs. none.	Not reported	Not reported	Not reported
Lee W Y W et al. (24)	OR 1.57 (95% CI 1.15-2.15) ref. Solid tumors.	**Overall leukemia:** Case Fatality Rate (CFR) 2.25, 1.13-4.57.	**Lymphoma:** CFR 1.63, 1.28-2.06.	**Multiple Myeloma:** CFR 2.03, 1.42- 2.83.
Pinato et al. (25)	Mean OS in HM patients with COVID-19: 47.6 (95% CI 6.9-34.1 days) vs. 86 days (95% CI 3.7-78.7) in the overall cohort of patients with cancer and COVID-19.	Not reported	Not reported	Not reported
Garcia-Suarez J. et al. (26)	The overall mortality rate of 33%. The median time from confirmation of COVID-19 to death was 9 days (IQR 5–18)	HR for mortality in patients with Hematologic malignancies and COVID-19. **AML** HR 2.22 (95% CI 1.31-3.74) **ALL** HR 1.52 (95% CI 0.36 -6.58) **CML** HR 0.37 (95% CI 0.08 – 1.70) **CLL** HR 0.92 (95% CI 0.56-1.51) ref. NHL	HR for mortality in patients with Hematologic malignancies and COVID-19. **HL** HR 1.20 (95% CI 0.56 – 2.58) ref. NHL	HR for mortality in patients with Hematologic malignancies and COVID-19. **Multiple Myeloma:** HR 0.80 (95% CI 0.49-1.28) ref. NHL
Vijenthira et al. (15)	Pooled risk of death: 34% (95% CI 28-39)	Pooled risk of death: **Acute Leukemias:** 41% (95% CI 30-52) **CLL:** 31% (95% CI 23-40)	Pooled risk of death: **Lymphomas, excluding CLL:** 32% (95% CI 18-48)	Pooled risk of death: **MPN:** 34% (95% CI 19-51)
Passamonti et al. (27)	The standardized mortality ratio of the overall study population of HM with COVID-19 was 2.04 (95% CI 1.77–2·.4 as compared to the overall Italian population with COVID-19	Mortality HR (95% CI) (ref. Myeloproliferative neoplasms) **AML** 3.49 (1.56-7.81) **ALL** 1.65 (0.46-5.94) **CLL** 1.64 (0.77-3.51)	Mortality HR (95% CI) (ref. Myeloproliferative neoplasms) **HL** 1.30 (0.36-4.66) **Indolent lymphomas** 2.19 (1.07-4.48) **Aggressive lymphomas** 2.56 (1.34-4.89)	Mortality HR (95% CI) (ref. Myeloproliferative neoplasms) **MDS** 1.58 (0.69-3.62) **Plasma cell neoplasms** 2.48 (1.31-4.69)
Booth et al. (28)	All-cause mortality 44% (Follow-up period for live patients was 24 days and 11 days for deceased patients)	All-cause mortality OR (95% CI) (ref. Lymphoma & Waldenstrom Macroglobulinemia) **ALL, AML & MDS combined** 2.12 (1.31-3.44) **CLL** 1.16 (0.69-1.93) **MPN & CML combined** 1.58 (1.13-2.23)	All-cause mortality OR (95% CI) (ref. all other subgroups combined) 0.64 (0.48-0.86)	All-cause mortality OR (95% CI) (ref. Lymphoma & Waldenstrom Macroglobulinemia) **Myeloma & plasmacytoma** 1.58 (1.13-2.23)
Rubinstein et al. (29)	30-day mortality in HM patients with COVID-19: 19%	30-day mortality in patients with HM subtypes having COVID-19: **AML** 16% **ALL** 12% **CML** 16% **CLL** 22%	30-day mortality in patients with HM subtypes having COVID-19: **High-grade NHL** 24% **Low-grade NHL** 16% **HL** 15%	30-day mortality in patients with HM subtypes having COVID-19: **MDS** 37% **MPN** 29% **Plasma cell dyscrasias** 19%
Visco et al. (30)	Not reported	Not reported	Overall, 100-day mortality in patients with Lymphoma having COVID-19: 23% (95% CI 20%-27%) ————— HR (95% CI) for OS (ref. LBCL) **HL** 0.30 (0.15-0.60) **i-NHL** (indolent NHL) 0.70 (0.50-0.99) **MCL** (Mantle Cell Lymphoma) 0.97 (0.55-1.73) **TCL** 1.29 (0.72-2.33)	Not reported
Regalado-Artmandi I et al. (18)	Not reported	Not reported	The overall mortality rate of 34.5% in patients with Lymphoma having COVID-19 ————— HR of death in **high-risk Lymphoma** was 2.8 (95% CI 1.2-6.1) (ref. low-risk) HR of death in **DLBCL** was 2.7 (95% CI 1.4-5.2) (ref. FL)	Not reported
Mato et al. (31)	Not reported	Not reported	**CLL:** Overall CFR 33%	Not reported

ALL, Acute Lymphoid Leukemia; AML, Acute MyeloidLeukemia; CFR, Case Fatality Rate; CLL, Chronic LymphoidLeukemia; CML, chronic Myeloid Leukemia; DLBCL, DiffuseLarge B-Cell Lymphoma; FL, Folicular Lymphoma; HL,Hodgkin’s Lymphoma; HM, Hematological Malignancies; HR,Hazard Ratio; i-NHL, indolent-Non-Hodgkin’s Lymphoma; IQR,Inter-Quartile Range; MCL, Mantle Cell Lymphoma; MDS,Myelodysplastic syndrome; MPN, Myeloproliferative Neoplasm;OS, Overall Survival; TCL, T-cell Lymphoma.

**Figure 7:**
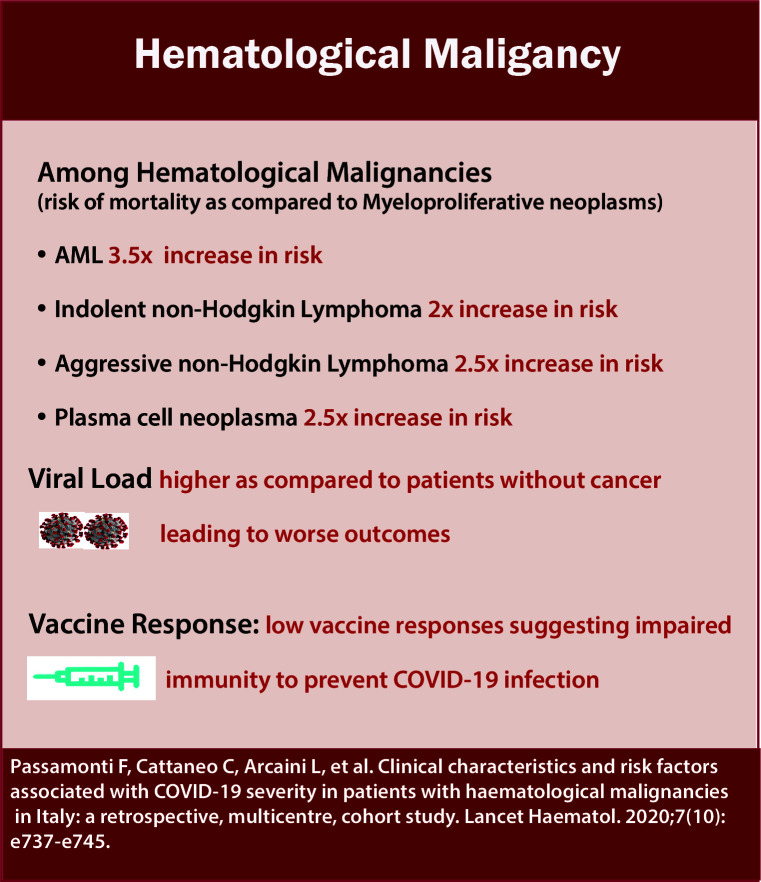
Box-6: Hematological malignancy.

Within the context of HM, some conditions predispose to worse outcomes (**Figure 7**/Box 6). In particular, acute leukemia, plasma cell neoplasms, and MDS have independently demonstrated worse outcomes in several reports (27, 28). Data from 757 patients with HM and COVID-19 from CCC19 showed around 37% of patients developed severe COVID-19 disease requiring mechanical ventilation or ICU admission or death. The rate of severe COVID-19 was highest in patients with chronic lymphocytic leukemia (CLL) (53%) and lowest in patients with Hodgkin lymphoma (23%). Unadjusted rates of severe COVID-19 outcomes were higher in patients receiving recent cytotoxic therapy. Patients receiving cellular therapy or transplant within a year of COVID-19 diagnosis had similar rates of severe COVID-19 and 30-day mortality compared to patients who had not received such therapies within a year (29). On comparing overall survival between T-cell Lymphoma (TCL) as compared to Low-grade B-Cell lymphoma (LBCL), no significant difference was observed (HR 1.29, 95% CI 0.72-2.33) (30).

**Figure 8:**
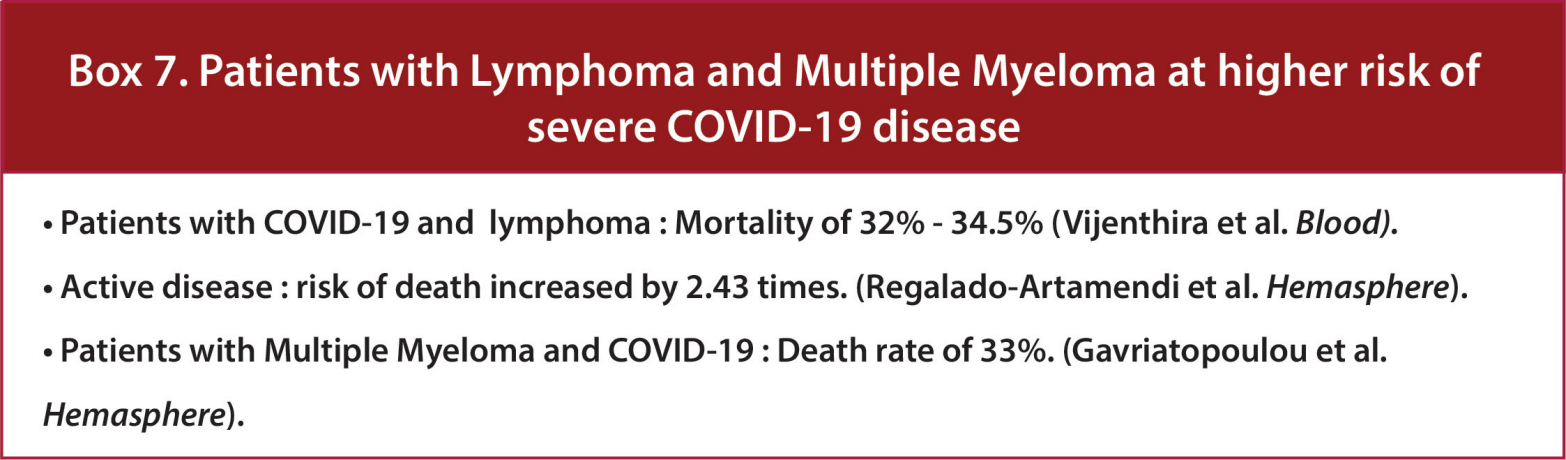
Box-7 Patients with Lymphoma and multiple myeloma at higher risk of severe COVID-19 disease.

In both a pooled analysis of 23 studies including 412 patients with multiple myeloma (MM) and a retrospective study conducted by the International Myeloma Working Group that included 650 patients with MM and Covid, the estimated risk of death due to COVID-19 was 33% (37) (**Figure 8**/Box 7). Similarly, a multicenter retrospective study of adult patients with COVID-19 and Lymphoma showed overall mortality of 34.5% among patients with active disease increasing the risk of death by 2.43 times. (HR 2.43, p=0.01) (18). Later, a meta-analysis of 34 studies also showed a mortality rate of 32% for patients with Lymphoma (15). In March 2021, a multicenter retrospective study from 19 centers in Madrid focusing on risk factors and mortality in COVID-19 and Lymphoma showed an overall mortality rate of 34.5% (18). Among lymphomas, reports have shown variable results. One study shows that Hodgkin lymphoma and aggressive non-Hodgkin Lymphoma are associated with worse COVID-19 outcomes (27), while another indicates lower mortality compared to other HMs (29).

**Figure 9:**
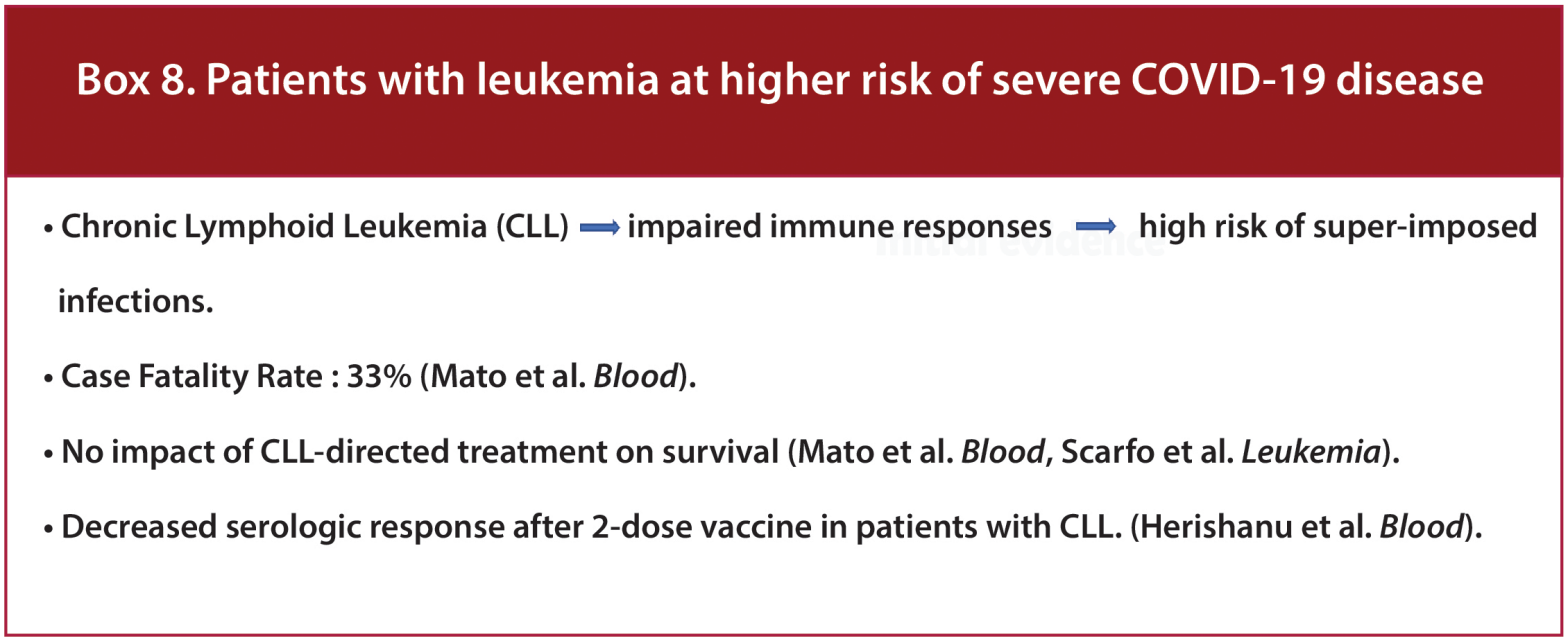
Box-8 Patients with leukemia at higher risk of severe COVID-19 disease.

Results of the UKCCMP cohort have shown that leukemia increased CFR compared to the rest of its cohort of cancer patients (2·25; p=0·023) (24). CLL is associated with impaired immune responses to common pathogens leading to a high risk of super-imposed infections (**Figure 9**/Box 8). This profound immune dysregulation is reflected by hypogammaglobulinemia, qualitative and quantitative B- and T-cell defects, including impaired response to vaccination, & CD4+ lymphopenia, innate immune dysfunction and neutropenia (38). In a multicenter study on patients with COVID-19 and CLL across 43 international centers, hospital admission occurred in 90% of patients, with a case fatality rate of 33%. Treatment naive (Watch-and-wait) and treated cohorts had similar rates of admission, ICU admission, intubation, and mortality with no impact of CLL-directed treatment with Bruton Tyrosine Kinase inhibitors (BTKi’s) at COVID-19 diagnosis on survival, suggesting that the subgroup of CLL patients admitted with COVID-19 were at high risk of death regardless of disease phase or treatment status (31). Similar results were observed from a multicenter European cohort study which showed that anti-leukemic treatment (particularly BTK inhibitors) did not increase mortality but rather appeared to exert a protective effect. Also, age and comorbidities did not impact mortality, alluding to the relevant role of CLL and immunodeficiency (39).

A study evaluating the prognostic significance of SARS-CoV-2 viral loads in patients with and without cancer demonstrated that patients with HM had higher median viral loads than patients without cancer, and high viral load was associated with an increased risk of in-hospital mortality. Patients with HM have low vaccine responses suggesting impaired immunity to prevent COVID-19 infection (33). On evaluating vaccine response, patients with CLL, in particular, had a lower serologic response after a 2-dose regimen of BNT162b2 mRNA COVID-19 vaccine as compared to sex- and age-matched healthy control subjects (52% vs. 100%, respectively; adjusted odds ratio, 0.010; 95% confidence interval, 0.001-0.162; P <.001) (40). Patients with cancer are also found to have an increased viral load. Ct (cycle threshold) values and rtPCR can be used as a marker to evaluate viral load. Ct is defined as the number of cycles required for the fluorescent signal to exceed background levels and is inversely proportional to the amount of target nucleic acid in the sample. It was found that the patients with HM who had received chemotherapy or targeted therapy within the previous six months had lower median Ct values than those who had not (22.6 vs. 28.8), indicating high viral load as compared to other patients (33).

Among the solid tumor malignancies, it is hypothesized that patients with thoracic cancer have worse outcomes based on the location and risk associated with COVID-19 pneumonia. Pre-existing lung injury can impact respiratory reserve and comprise outcomes in patients. CCC-19, in their updated analysis including 2749 patients with cancer, demonstrated that the mortality rate in patients with lung cancer (n=237) was higher (26%, n=61/237) as compared to the overall cohort (16%, n=433/2749) (41). Similarly, studies from DOCC (Dutch Oncology COVID-19 Consortium) and GCO-002 CACOVID (France) have shown that amongst solid tumors, lung or thoracic primary tumor sites were independently associated with COVID-19 severity and mortality (17, 42). TERAVOLT registry exclusively evaluated patients with thoracic cancers (43). On multivariate analysis of 1012 patients with active thoracic cancer, age ≥ 65 (OR 1.53 CI 1.11-2.1), active smoking (OR 2 CI 1.3-3), higher stage of cancer (OR 1.9 CI 1.3-2.7), ECOG PS ≥2 (OR 3.7 CI 2.7-5), steroids prior to COVID diagnosis (OR 1.8 CI 1.2-2.7), were associated with increased risk of death, while chemotherapy and TKI therapy use were not and interestingly patients on immunotherapy appeared to be at a decreased risk for mortality (OR 0.6 CI 0.5-0.97). Data on COVID-19 risk in patients with secondary lung metastases instead of primary thoracic tumors have been incompletely characterized. However, it is hypothesized that such individuals would be negatively impacted by COVID-19 disease.

While not a malignancy, it is also found that patients with benign hematologic conditions, particularly individuals with Sickle Cell disease, are at higher risk of severe COVID-19 infection as compared to the general population (44). Findings by Mucalo et al. show that individuals with SCD who have COVID-19 infection have higher rates of death due to COVID-19 than the general Black population. Young adult SCD patients aged 18-34 years had a case fatality rate of 3.3%, and those aged 34-50 years had a rate of 14.9% (45).

### 3.5 Type of cancer therapy

Patients with cancer receive various types of systemic therapies with different mechanisms of action, toxicity profiles, and outcomes. These therapies range from hormonal, targeted, cytotoxic chemotherapies, and immunotherapies, including cellular-based treatments. Several reports have investigated the impact of systemic therapy type on COVID-19 outcomes (13, 21, 24).

A comprehensive review of the CCC-19 registry evaluating the differential impact of different treatments in 4,966 patients with cancer and COVID-19 disease demonstrated that receipt of cytotoxic chemotherapy within 3 months of COVID-19 diagnosis was associated with worse COVID-19 disease severity [OR 1.28 (1.04 – 1.58) and 30-day mortality [OR 1.61 (1.15 – 2.24)]. Amongst cytotoxic regimens, R-CHOP-like, platinum combined with etoposide, and DNA methyltransferase inhibitors were associated with the highest 30-day all-cause mortality (13). While the CCC-19 registry represents the most comprehensive dataset, previous datasets have demonstrated mixed results on the risk associated with various cancer-directed systemic therapies. Initial reports from the pandemic were limited by modest statistical power, with a limited number of chemotherapy patients (21, 46, 47). However, a meta-analysis of 16 studies including 4,510 cancer patients showed that COVID-19 patients receiving chemotherapy within the 30 days preceding a COVID-19 diagnosis were at increased mortality (OR 1.85, 1.26-2.71) after adjusting for confounding variables (48).

**Figure 10:**
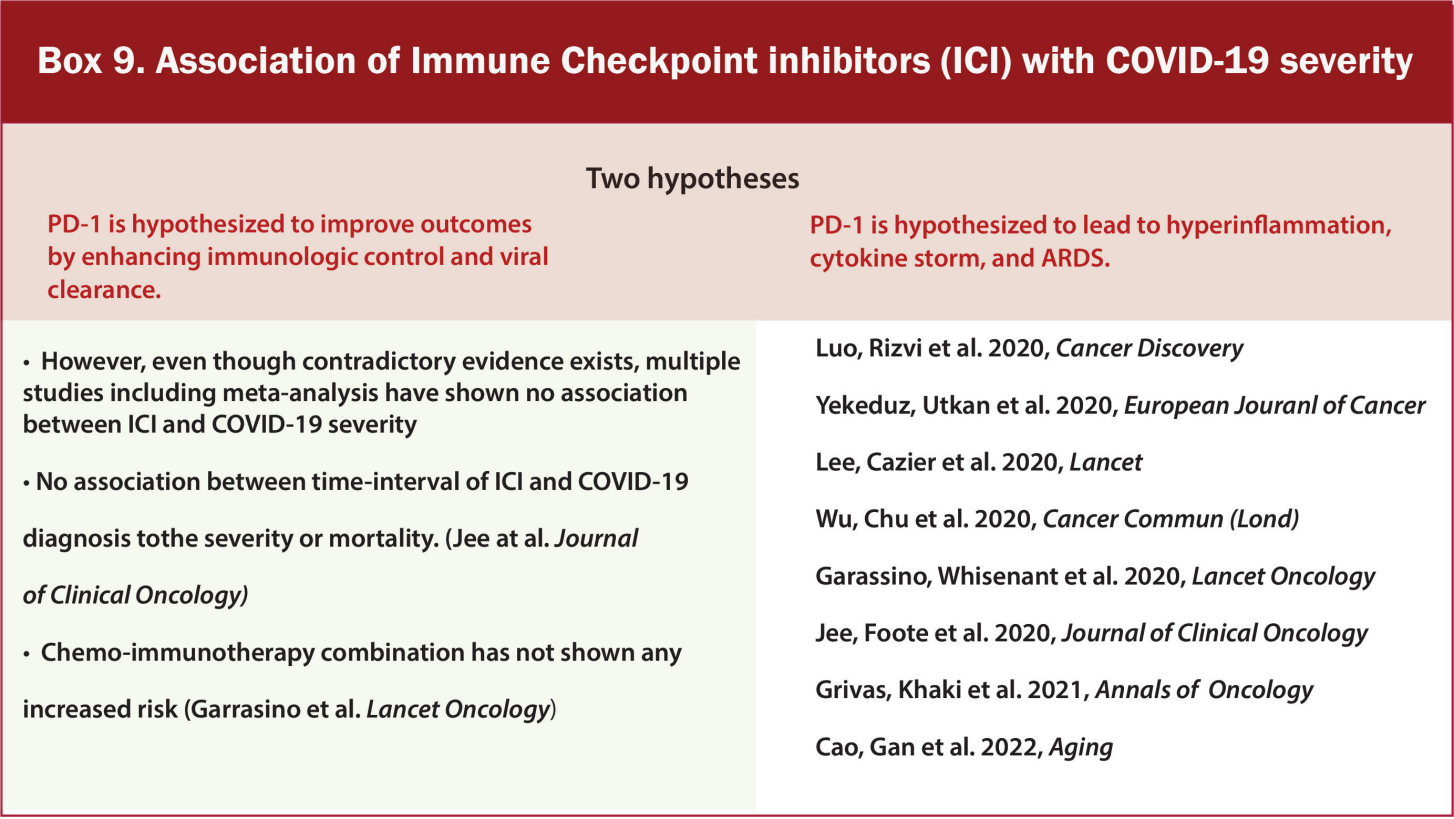
Box-9 Association of Immune Checkpoint Inhibitors (ICI) with COVID-19 severity.

Similarly, there have been mixed results regarding the impact of immunotherapy on COVID-19 outcomes in cancer patients having COVID-19 (**Figure 10**/Box 9). Immunotherapy poses a peculiar risk. On the one hand, PD-1 is hypothesized to improve outcomes by enhancing immunologic control and viral clearance, whereas, on the other hand, it can lead to hyperinflammation, cytokine storm, and ARDS (49, 50). In a comprehensive report evaluating the role of cancer therapy type on outcomes in patients with COVID-19 and cancer, there was no significant association with mortality among patients who had received immunotherapy in the past 3 months vs. those who did not receive any therapy (13). Contradictory evidence was seen in one of the reports. On a subgroup analysis from 17 studies comprising 3581 cancer patients, patients who had received immunotherapy within 90 days showed an increased risk of exacerbation (OR 2.53,95%1.30–4.91, P = 0.006) (51). However, another study from the UK of 800 COVID-19 patients with active cancer: compared with patients who were not on any therapies, patients on immunotherapy (n=44; OR 0·59 [95% CI 0·27–1·27]; p=0·177) did not have increased risk of severity or COVID-19 (47). Another study included 69 patients and defined 5 groups according to the interval from the last ICI dose to COVID-19 diagnosis: no prior PD-1 ever received PD-1; last dose within 6 months, 6 weeks, and 3 months. Overall, there was no statistically significant difference in the different groups regarding the rate of hospitalization, severe disease, or death. Overall, there was no significant difference in prognosis regardless of PD-1 blockade exposure (49). At the same time, Wu et al. observed a similar severity risk in different groups based on the last dose of immunotherapy. Even though data showed that patients who received 3 or more cycles of immunotherapy were more likely to develop severe COVID-19, no statistical significance was observed (52). In the initial report of 423 COVID-19 and cancer patients by MSKCC, 31 patients had received an Immune Checkpoint inhibitor (ICI). They observed a significant association of Immunotherapy as a risk factor for severe outcomes in ICI-treated patients, independent of age, cancer type and other co-morbidities (53). However, in a follow-up report focusing on PD-1 blockade therapy from the same center, the same findings were disproven after adjustments for smoking status from the same group of patients on ICI (n=41) (49). An initial report from the global consortium (TERAVOLT), including a total of 200 patients with COVID-19 and thoracic cancers from eight countries, showed no impact on the risk of death for chemotherapy–immunotherapy and ICI alone (43). No association was observed between the time interval of the last ICI administration and COVID-19. Treatment with immunotherapy within 35 days was not associated with the primary end point of severe or critical COVID-19 event (HR, 1.80; 95% CI, 0.89 to 3.50; P = 0.11), although the number of patients who received immunotherapy was small (n = 18). When treatment administration windows were adjusted to 14- or 90-day intervals from COVID-19 diagnosis, the absence of association between chemotherapy or immunotherapy treatment and COVID-19 severe or critical infection was preserved (21).

In a retrospective review of 1545 patients from the Massachusetts Department of Public Health (DPH) data with cancer treated with ICI, there was no difference in mortality of patients receiving ICI. However, both the patients who were treated with combination therapy died, which may suggest that monotherapy should be used in patients at high risk of COVID‐19 mortality when clinically reasonable (54). It was also found that the patients receiving dual blockade therapy had significantly higher hospital admission and mortality rates (54, 55). In a meta-analysis of 16 studies, univariate analyses of immunotherapy within the last 30 days before COVID-19 diagnosis did not increase the risk of severe disease and death in cancer patients (OR:1.60; 95% CI:0.72–3.52; p = 0.25 and OR:1.12; 95% CI:0.60–2.08; p = 0.72, respectively) (48). A recent meta-analysis of 20 studies focusing on the safety of administering immunotherapy in cancer patients with COVID-19 also showed no association with increased mortality in cancer patients receiving ICI within 30 days before the diagnosis of COVID-19 (56). Even though results have been variable, most studies above have concluded that checkpoint inhibitor treatment appears relatively safe in patients with COVID-19 and cancer irrespective of the number of cycles, duration or interval of last ICI administration and COVID‐19 (21, 47–49).

With regards to other immunologic treatments such as bone marrow transplant, CAR-T, or other cellular therapies, available data demonstrates that individuals diagnosed with COVID-19 after CAR-T-cell therapy experienced lower survival (57, 58), however robust data on the impact of other cellular therapies are limited. Furthermore, receipt of CAR-T cell therapy is found to have significantly lower vaccine responses (59).

Across the board, hormonal/endocrine therapy has not demonstrated adverse COVID-19 outcomes (13, 47). Furthermore, given the role of the androgen-regulated gene TMPRSS2 in SARS-CoV-2 pathogenicity, it was hypothesized that androgen deprivation therapy in men with prostate cancer might have a protective effect on COVID-19 outcomes. An analysis of 1106 men with prostate cancer demonstrated no association between androgen deprivation therapy and mortality (60).

Regarding the outcomes of targeted therapy, the data have been limited. To date, many prominent studies have shown no impact of targeted therapy on mortality (13, 21, 47). Sensitivity analysis by Lee et al. from 309 cancer patients from MSKCC demonstrated that receipt of targeted therapy within 35 days was associated with a trend toward increased risk of ICU stay or death after diagnosis of COVID-19 (HR, 2.2; 95% CI, 1.1 to 4.3; P = .02). Similarly, the UKCCMP study, including patients with HM, showed no association between anti-CD19/anti-CD20 treatment and increased risk of death (28). Further research focusing on targeted therapies and their impact on outcomes of COVID-19 is needed to clarify further the risk associated with treatment.

Additional therapeutic strategies integrate radiation therapy and also surgery. Data are more limited on the impact of such treatment strategies on COVID-19 outcomes. In summary, patients with cancer who were receiving cytotoxic chemotherapy were found to be at higher risk of COVID-19-related outcomes. In contrast, other therapies, including immunotherapy, hormonal therapy, and targeted therapy, were safe overall. However, further studies are needed to evaluate their impact.

### 3.6 Comorbidities

Multiple series independent of underlying cancer status have demonstrated increased mortality risk in patients with underlying hypertension, diabetes, pulmonary comorbidities, and renal and cardiovascular co-morbidities. There is a relationship between the number of comorbidities and associated COVID-19 risk (47). Similarly, studies in patients with cancer and COVID-19 have evaluated the effect of comorbidities defined by the Charlson comorbidity index (CCI) and their impact on COVID-19 outcomes (61, 62). The CCI includes a global assessment of comorbidity status, including 14 different comorbidities (63). Higher CCI scores have shown worse outcomes in patients with cancer and COVID-19 (62, 64). Many studies have demonstrated that patients with pre-existing pulmonary conditions, including COPD and asthma, are at risk of worse COVID-19 outcomes, possibly due to increased inflammatory lung damage leading to decreased pulmonary reserve (13, 43, 64).

### 3.7 Smoking

Smoking has many detrimental effects on immune and lung function. Like pulmonary comorbidities, smoking can reduce pulmonary reserve due to damage to airways and parenchyma, potentiating harmful effects of COVID-19. Studies have evaluated the impact of smoking by categorizing patients as current, former, or never smokers. Smoking status has been demonstrated to be an independent factor for mortality in COVID-19 patients (23). Grivas et al. showed that smoking history was significantly associated with increased 30-day mortality (13). Other studies have confirmed these results (34, 43). Multivariate analysis from the TERAVOLT registry demonstrated that smoking history among 200 patients with thoracic cancers was the only variable associated with increased mortality risk (HR 3.18, 95% CI 1.11 – 9.06) (43). Additionally, smoking use increased the risk of post-COVID sequelae (65).

### 3.8 Risk assessment

As new data are available on risk factors associated with COVID-19 disease severity in cancer patients, risk stratification tools to aid clinicians in predicting prognosis in patients with COVID-19 and cancer are limited. A developing resource is the SMART COVID Navigator, a clinical decision support tool that allows physicians to predict the fatality and severity of COVID-19 progression given a patient’s medical conditions (66). CCC-19 has also developed a Geriatric risk assessment index that integrates age, modified CCI, and ECOG performance status with COVID-19 severity and 30-day mortality among older adults with cancer. On utilizing this CCC19 geriatric risk index on 5671 patients with cancer and COVID-19, it was found to be strongly associated with COVID-severity and 30-day mortality (67). Given the heterogeneity of competing risk factors in patients with cancer, individualized risk assessment is critical to predicting the risk of adverse outcomes associated with COVID-19 in patients with cancer. Such information will be important in developing personalized strategies to prevent COVID-19 disease development and prioritize treatments in patients diagnosed with COVID-19.

## 4 Prevention

In the high-risk cancer population, prevention has been critical to curbing the impact of the pandemic on patients with cancer. Efforts have been made at the patient, health system, and population levels to mitigate the effects of the ongoing outbreak for patients with cancer. This includes (1) screening, barrier and isolation strategies (2), Masking/PPE (3), vaccination, and (4) systemic therapies (e.g., evusheld) to prevent disease onset in patients.

### 4.1 Barrier and isolation strategies

Health systems have adapted to the pandemic and developed mitigation strategies to optimize care delivery for patients with cancer. Data from the ASCO registry demonstrated significant changes across oncology practices in response to the pandemic. The pandemic has catalyzed the expansion of telehealth practices (68). While this has had several positive effects on clinical practice, limitations exist, including integration with translator service, application in rural areas with limited internet access, application for older adults, and ensuring technical support for clinicians and patients (65, 69). Clinical practices have employed 1) symptom screening for both staff and patients before entry into clinical spaces, 2) asymptomatic COVID-19 testing before receipt of antineoplastic treatment, procedures/surgeries, or receipt of radiation treatment (69), and 3) visitor policies to restrict high volumes in clinical spaces. Furthermore, the deployment of physical barriers and restrictions in clinician works spaces have been established to limit transmission.

At the population level, social distancing measures were implemented to mitigate the risk of COVID spread. During this pandemic, many other interventions played an impactful role, including school closures, senior isolation, limiting social gatherings, “work from home strategies” for non-emergency workers, case isolation, restriction on travel, and border closures in select countries. The changes in contact matrices implemented during the lockdown reduced the number of contacts by 81% compared to baseline mixing patterns (70). Additional studies have demonstrated that social distancing and isolation measures are beneficial in decreasing mortality and overall economic expenditure related to COVID-19 (71, 72).

### 4.2 Masking and personal protection equipment

Since the pandemic’s beginning, strict masking and Personal Protection Equipment (PPE) policies have been enforced in oncology practices. The availability of PPE and the incidence of Covid-19 cases in different regions made these policies dynamic and different in different cancer centers. In general, all healthcare providers and patients are still required to wear a face mask while inside the centers (73).

### 4.3 Vaccination

The urgency of this pandemic was a catalyst in the global research community for rapid vaccine development against COVID-19. Two mRNA vaccines (BNT162b2 Pfizer-BioNTech COVID-19 Vaccine [Comirnaty] and Moderna COVID-19 Vaccine [Spikevax] have received full FDA approval (74). Each of these vaccines requires a series of two injections three or four weeks apart. At present, vaccination is recommended for all individuals over the age of 6 months. None of the trials reporting on the efficacy of the vaccines were explicitly conducted on cancer patients. Several trials had partial exclusion criteria for cancer patients, particularly those receiving anticancer therapy. For example, in NCT04470427 and NCT04368728 trials for mRNA-1273-P301 (Moderna vaccine) and BNT162b2SA (Pfizer & BioNTech vaccine), respectively, immunosuppressed or deficient patients, as well as those who had received systemic immunosuppressants at specific time intervals, were initially excluded from enrollment. Nonetheless, vaccination has proven to be an effective strategy for mitigating disease severity in an individual patient and viral spread within the community. Over time, vaccine efficacy has been eroded by two factors. The first is that immunity to coronaviruses (whether induced by vaccines or natural infection) wanes over time and breakthrough and/or reinfection become increasingly common 4 – 6 months after the last exposure to the virus or to vaccination. In addition, SARS CoV-2 has been continuously since its introduction into humans in 2019 and antibodies raised by the initial vaccines are much less capable of inducing neutralizing antibodies to the currently circulating Omicron variants. Because of these two factors, it has become clear that it will be necessary to boost immunity periodically and that the “booster” vaccines will need to be tailored to induce immune responses to contemporaneously circulating strains. At this writing both Moderna and Pfizer have developed “hybrid” vaccines that include mRNA that induces expression of two distinct spike proteins. One is similar to the originally circulating strains and the other is based on an early Omicron strain. The CDC recommends that those five and older receive one of the two hybrid vaccines as a booster if it has been at least two months since their initial vaccine series (75).

From these phase 3 studies, data has evolved regarding vaccination in patients with cancer. A study evaluating seroconversion to SARS-CoV-2 spike (S) protein in patients with cancer following the first vaccination with the BNT162b2 vaccine showed that a single vaccine dose failed to induce seroconversion in most patients with cancer. On boosting the patients at day 21, 95% of patients with solid cancers were seropositive compared to 30% who were not boosted. Even though low, seropositivity increased from 11% in non-boosted patients with HM to 60% in boosted patients. The response was 100% on boost in the healthy population (76). Similarly, lower seroconversion rates were observed in patients with CLL (40) and immunosuppressed individuals, including patients with cancer (77). While patients with cancer had lower rates of pre-booster seroconversion, post-booster seroconversion at 4-6 months was 94% for patients with HM and 100% for patients with solid tumors (78). While seroconversion may not directly equate with immunity, these studies suggest blunted vaccine response in immunocompromised individuals with cancer and provide a rationale for booster vaccination in immune-compromised individuals. CCC-19 recently reported data on breakthrough COVID-19 infections in 54 fully and 77 partially vaccinated patients. These patients had substantial comorbidities with a higher representation of patients with HM. Lymphopenia, a strong risk factor for worse outcomes, was present in 46% of fully vaccinated patients and 28% of unvaccinated patients (79).

### 4.4 Systemic therapy

Prior to the emergence of the Omicron variant of SARS CoV-2 in late 2021, the FDA had issued a series of emergency use authorizations (EUA) for long-acting monoclonal antibodies, such as tixagevimab plus cilgavimab (Evusheld) for pre-exposure prophylaxis COVID-19 in certain adults and pediatric individuals (80). As shown by different studies, immunocompromised patients have a low immune response to vaccines (76–78). Evusheld was therefore authorized for non-infected individuals with either moderate to severely compromised immune systems due to underlying medical conditions or taking immunosuppressive medications or treatment that may not respond adequately to vaccination (81). It was also authorized for those individuals who are not recommended to get COVID-19 vaccination due to a history of severe adverse reactions to a COVID-19 vaccine and/or component(s) of those vaccines. Of concern, laboratory studies demonstrated lower levels of neutralizing activity for evushield and all other commercially available monoclonal antibodies to SARS CoV-2 against the more recently circulating SARS-CoV-2 variants (82). At this writing there are no FDA approved monoclonal antibodies for the prevention or treatment of SARS CoV-2.

## 5 Treatment

**Figure 11:**
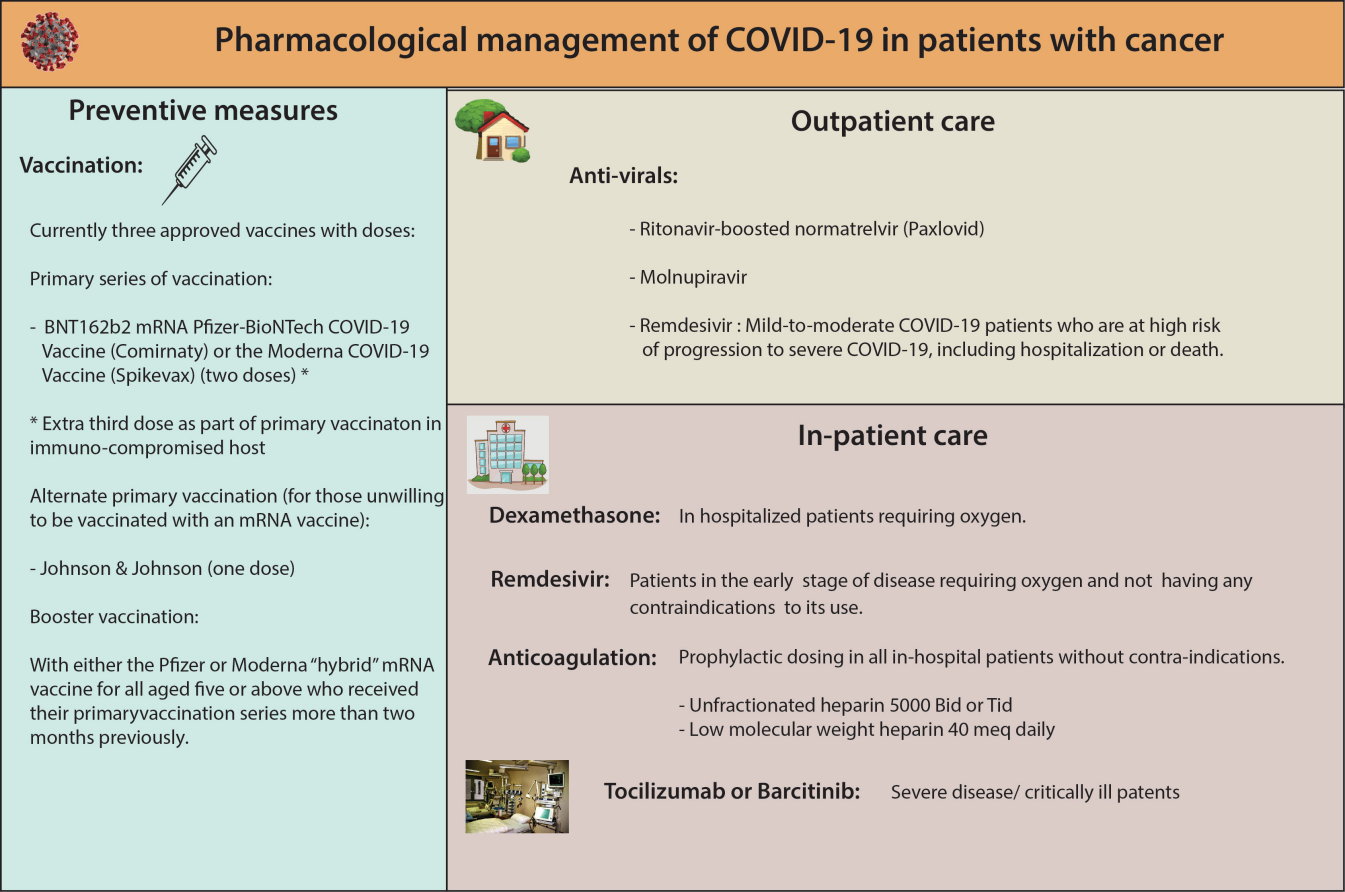
Pharmacological management of COVID-19 in patients with cancer.

COVID-19 management continues to evolve as more data on optimal treatment strategies and additional therapies are available to mitigate risk in patients with COVID-19 and cancer (**Figure 11**).

### 5.1 Outpatient management

Several neutralizing antibodies have been investigated for use in outpatients with COVID-19, including individuals with cancer. Several randomized, placebo-controlled trials of non-hospitalized patients with mild to moderate COVID-19 symptoms and certain risk factors for disease progression demonstrated that the use of anti-SARS-CoV-2 monoclonal antibody products reduced the risk of hospitalization and death (83–86). Most of these trials had a very low presentation of immunocompromised patients (1.5% in bamlanivimab + etesevimab, 0.7% in casirivimab + imdevimab, not specified in sotrovimab) (83–86). A retrospective review of 3596 high-risk patients receiving bamlanivimab or casirivimab-imdevimab showed a hospitalization rate of 8.4% in immunosuppressed patients compared to immunocompetent (3.2%) (OR=2.78, 95% CI 1.91-4.00) (87). Recently, with the emergence of the omicron variant, the activity of the previously effective monoclonal antibodies has been so substantially eroded that they no longer provide protection against infection and they are no longer authorized for this indication by the FDA (88). Although efforts are underway to develop “universally” neutralizing monoclonal antibodies, none have yet been delineated, and the plasticity of the viral spike protein makes this a tall order.

With the decline in efficacy of neutralizing monoclonal antibodies to SARS CoV-2, therapeutic efforts are increasingly shifting to antiviral agents. Two oral antiviral agents are available for the high-risk patient population, including cancer: ritonavir-boosted nirmatrelvir and molnupiravir (89, 90). Nirmatrelvir is active against a viral protease that plays an essential role in viral replication, demonstrating antiviral activity against all coronaviruses known to infect humans. It is packaged with ritonavir, a robust boosting agent (91, 92). Molnupiravir is a prodrug form of mutagenic ribonucleoside and results in viral mutation and lethal mutagenesis, leading to potent antiviral action (93). A phase three trial of molnupiravir significantly lowered hospitalization or 29-day mortality rates in initially non-hospitalized, unvaccinated adults with mild-to-moderate COVID-19 illness (94). However, only 2% of patients had active cancer in this trial. A separate phase three trial of ritonavir-boosted nirmatrelvir demonstrated a larger reduction in hospitalization or death than that observed in the molnupiravir trial, and it is favored as the first-line choice in patients who do not have medical contraindications to the use of the drug (95). Drug-drug interactions with ritonavir represent the most important challenge to using ritonavir-boosted nirmatrelvir. Several sources are available to assist in identifying and managing these interactions (96, 97). Although it can be logistically challenging, patients in higher risk categories for severe disease outcomes which cannot be treated with ritonavir-boosted nirmatrelvir should be offered intravenous remdesivir as a three-day series of outpatient infusions (98).

### 5.2 Inpatient management

For hospitalized patients, data from randomized controlled trials (ACTT, RECOVERY, Solidarity) support the use of remdesivir, dexamethasone, and tocilizumab or barcitinib in COVID-19 patients admitted to the hospital (99–101). Among all these treatment modalities, dexamethasone has resulted in lower 28-day mortality among those receiving invasive mechanical ventilation or oxygen alone (101). Except for remdesivir, other treatments, including hydroxychloroquine, lopinavir, ivermectin, and interferon beta-1, have not shown any effect on mortality, hospitalization, or initiation of ventilation (100, 102, 103). None of these trials reported the presence or breakdown of cancer patients.

Patients with COVID-19 are also at risk for cytokine storm. Currently, barcitinib and tocilizumab are approved to mitigate symptoms and organ damage associated with cytokine storm. Baricitinib, a Janus kinase (JAK) inhibitory used to treat rheumatoid arthritis, has immunomodulatory properties and potential antiviral effects through the impediment of viral entry. Tocilizumab is a monoclonal antibody against interleukin-6 receptor-alpha, which binds to both membrane and soluble IL-6 receptors, reducing inflammation. It has been used to treat chimeric antigen receptor (CAR) T cell-induced cytokine release syndrome in cancer patients (104, 105). Given the role of IL-6 in COVID-19-associated lung damage, it has been used in this clinical scenario (106).

### 5.3 Anticoagulation

COVID-19 has been associated with thrombosis, disseminated intravascular coagulation (DIC), and cytokine storm. Guidelines for the use of coagulation are constantly evolving. Various risk assessment tools like Padua, IMPROVE, or caprine are available to evaluate VTE risk in COVID-19 and cancer patients (107, 108). Along with anti-COVID-19 management, anticoagulation has become an integral part of the management. Anticoagulation can not only prevent thrombosis but can also delay or even stop the progression to DIC. Consensus guidelines from the American Society of Hematology, NIH, NICE, and WHO recommend initiating standard prophylactic dosing in all in-hospital patients without contraindications (109–111).

## 6 Discussion

Adaptability at the patient, health system, and community levels will be the key to mitigating risk for patients with cancer during the COVID-19 pandemic. Throughout the pandemic, isolation and masking strategies have evolved to mitigate risk. The vaccination rate has increased significantly. With more than 559,436,368 doses administered in the US, at least 77% of the population has received at least one dose, with 65% fully vaccinated by March 2022 (112). Different cancer societies have published guidelines for efficient cancer-care delivery during this pandemic (113, 114). Cancer practices will continue to adapt as newer guidelines and practice policies evolve throughout the pandemic.

With new changes, there exist many challenges. During the earlier phase of the pandemic, many clinical and research trials were halted. Translational research studies dependent on specimen procurement were also profoundly impacted. Furthermore, funding has shifted towards COVID-19 mitigation strategies, vaccine development, and treatment support. However, more funding allocations will be needed for future research (115). While the majority of available data on cancer patients stems from retrospective series, a key registry is the NCI CAPS prospective registry. Accrual was completed on 2/2022, and results are eagerly waiting. This data registry included prospective data capture to evaluate risk factors for severe disease, effects of COVID-19 on cancer therapy, Quality of Life-related outcomes, and a blood specimen collection for future biomarker studies (116).

The current impact of the COVID-19 pandemic on cancer care in the United States has resulted in decreases and delays in identifying new cancers and the delivery of treatment. In March-July 2020, compared to the baseline period of March-July 2019, there was a substantial decrease in cancer screenings, visits, therapy, and surgeries, varying by cancer type and service site. At the pandemic’s peak in April 2020, screenings for breast, colon, prostate, and lung cancers were decreased by 85%, 75%, 74%, and 56%, respectively, within parallel declines in oncologic surgical procedures and treatments. Along with the significant missed diagnosis, studies have also shown stage migration, with a higher proportion of patients presenting at a later stage of the disease (117–119). There is concern that the next 5-10+ years may show an increased incidence of late-stage disease with flattening or worsening cancer mortality as a result of decreased screening during the pandemic (120, 121). Innovative strategies will be needed to continue to ensure safe and effective cancer care delivery and screening strategies during the aftermath of the pandemic. Possibilities include home cancer screening, community-based screening, and other approaches (122).

**Figure 12:**
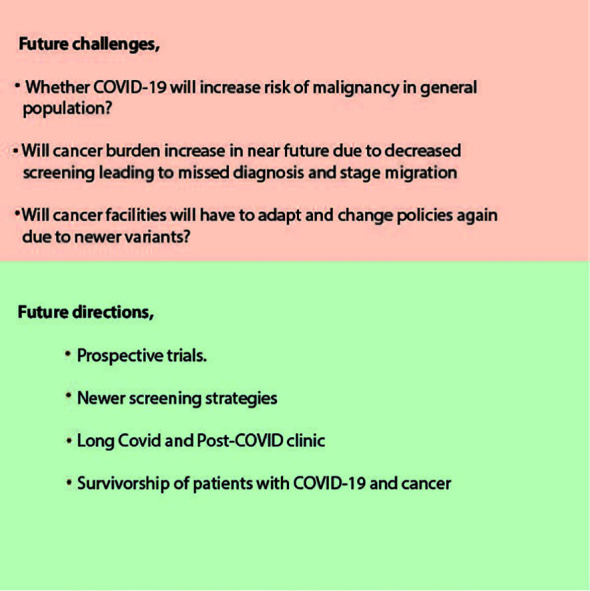
Future challenges and directions.

Post-COVID syndrome or long COVID sequelae are a real concern for patients with cancer. Healthcare practices worldwide have started establishing post-COVID clinics to tackle this mammoth task of understanding this survivorship burden. Long-COVID is associated with not only increased hospitalization for COVID-19 and complicated COVID-19 but also with an increased risk of death (65). It is observed that Long-Covid occurs in 60% of cancer patients, and it may persist up to 14 months after acute illness (123). Future investigation will guide us in deciding expectant management and surveillance imaging in these Long-COVID with cancer patients (**Figure 12**). Whether COVID-related CT changes will affect restaging or regular surveillance scans is still to be studied. At cancer centers, we will have to draw parallels between the journey of long COVID and many cancers while evaluating their common psychosocial, physical, lifestyle, and financial burdens (124). Also, the future will tell us whether COVID-19 will pose any increased malignancy risk in the general population.

## 7 Conclusion

The COVID-19 pandemic has had a dramatic impact on care delivery for patients with cancer. Ongoing collaboration between clinicians, researchers, health system administrators and policymakers will be important to ensure the optimization of care delivery strategies for patients with cancer. Creative patient-centered solutions are going to be critical in the years to come, post the pandemic.

## Author contributions

CJ, RM, and SL contributed to the conception and design of the review. CJ and RM wrote the first draft of the manuscript. CJ, RS, and RM wrote sections of the manuscript. RS, RM, and SL performed a critical review and editing of the final draft. RM and SL are the corresponding authors. All authors contributed to the article and approved the submitted version.
